# Chronic kidney disease in immigrant children: an emerging clinical-epidemiological scenario in clinical practice

**DOI:** 10.1590/2175-8239-JBN-2021-0217

**Published:** 2021-11-26

**Authors:** Mauro Oliveira Santos

**Affiliations:** 1 Escola Bahiana de Medicina e Saúde Pública, Salvador, BA, Brasil.; 2 Hospital Ana Nery, Salvador, BA, Brasil.; 3 Universidade Federal da Bahia, Salvador, BA, Brasil.

Dear editor,

Congratulations to the authors and editor of the original article with descriptive and analytical aspects of the sociodemographic, etiological and therapeutic profile addressing chronic kidney disease in immigrant children cared for in a center in Turkey, published in the Brazilian Journal of Nephrology, for addressing a theme a lot, since, considering the current political and economic scenario, it will be increasingly prevalent in clinical practice in different countries.

According to UNICEF^1^ (United Nations International Children's Emergency Fund), in some countries almost half of the refugee population are children. Studies have been published to inform about the main health problems in the refugee population. In a retrospective multicenter study, also carried out in Turkey that included the population aged 0 to 18 years, Balat et al. (2021)^2^ evaluated data from 633 children in 22 pediatric nephrology centers. The most common causes of kidney disease were congenital anomalies of the kidneys and urinary tract (CAKUT) (31.0%) and glomerular diseases (19.9%). The most frequent causes of CAKUT were non-obstructive hydronephrosis (23.0%) and vesicoureteral reflux (VUR) (18.4%). Of the patients undergoing a therapy program for chronic kidney disease (n=58), 53.4% ​​were on hemodialysis. Of the patients on peritoneal dialysis, 74% were on continuous ambulatory peritoneal dialysis (CAPD).

Also, in a study with the population of refugee children in Syria, Kara et al. (2017)^3^ evaluated 130 patients aged 1 month to 18 years in the Department of Pediatric Nephrology at the University of Gaziantep between September 2012 and January 2015. Of the 130 children evaluated, 59.6% were female, with a mean age of 7 ± 4 years, presenting as main causes CAKUT (26.2%) and nephrotic syndrome (18.5%), with 23.1% of patients with the disease being registered as chronic kidney disease. In this cohort, among the CAKUT, there was a higher prevalence of neurogenic bladder (35.3%), followed by VUR (17.6%) and obstruction of the ureteropelvic junction (17.6%).

The topic addressed by Mehtap Çelakil e Yasemin Çoban (2021)^4^ represents a relevant and emerging problem that highlights the importance of population migrations in understanding epidemiological and clinical factors that are important for diagnostic and therapeutic decisions. From the knowledge obtained by studies in Turkey, we can create horizons for research and search for knowledge to understand the immigrant population in Brazil in the last 10 years.

## References

[1] United Nations International Children's Emergency Fund (2019). Global annual results repost 2020: humanitarian action.

[2] Balat A, Kilic BD, Aksu B, Kara MA, Buyukcelik M, Agbas A (2021). Kidney disease profile and encountered problems during follow-up in Syrian refugee children: a multicenter retrospective study. Pediatr Nephrol.

[3] Kara MA, Kılıç BD, Çöl N, Özçelik AA, Büyükçelik M, Balat A (2017). Kidney disease profile of Syrian refugee children. Iran J Kidney Dis.

[4] Çelakıl M, Çoban Y (2021). Etiologic-sociodemographic assessment and comparison of dialysis modalities in pediatric Syrian migrants with chronic kidney disease. Braz J Nephrol.

